# Genetics and genomics of alcohol sensitivity

**DOI:** 10.1007/s00438-013-0808-y

**Published:** 2014-01-07

**Authors:** Tatiana V. Morozova, Trudy F. C. Mackay, Robert R. H. Anholt

**Affiliations:** Department of Biological Sciences and W. M. Keck Center for Behavioral Biology, North Carolina State University, Box 7617, Raleigh, NC 27695-7617 USA

**Keywords:** Addiction, Behavioral genetics, Genome-wide association, Quantitative trait loci, Meta-analysis

## Abstract

Alcohol abuse and alcoholism incur a heavy socioeconomic cost in many countries. Both genetic and environmental factors contribute to variation in the inebriating effects of alcohol and alcohol addiction among individuals within and across populations. From a genetics perspective, alcohol sensitivity is a quantitative trait determined by the cumulative effects of multiple segregating genes and their interactions with the environment. This review summarizes insights from model organisms as well as human populations that represent our current understanding of the genetic and genomic underpinnings that govern alcohol metabolism and the sedative and addictive effects of alcohol on the nervous system.

**Electronic supplementary material:**

The online version of this article (doi:10.1007/s00438-013-0808-y) contains supplementary material, which is available to authorized users.

“Alcohol is the anaesthesia by which we endure the operation of life”.

George Bernard Shaw

## Introduction

Alcoholic beverages have been around since time immemorial and have served economic, social, medical and religious purposes. Alcohol is unique among substance abuse drugs, as it is a natural by-product of fermentation. Moderate drinking of alcohol may offer health benefits (Marugame et al. 2007), including reduction in cardiovascular disease (Baer et al. 2002), ischemic strokes (Zeng et al. 2012; Peng et al. 2013), stress levels, incidence of type II diabetes (Conigrave et al. 2001; Koppes et al. 2005) and gallstone disease (Leitzmann et al. 1999). Excessive alcohol consumption, however, is a common cause of preventable death in most countries, and imposes a major socioeconomic burden. Alcohol abuse and dependence disorders are associated with marital instability, violent crime, fatal accidents, and injuries (Chick 2011). Heavy drinking is associated with increased risk of different types of cancer (Nelson et al. 2013; Touvier et al. 2013), higher cardiovascular disease mortality (Graff-Iversen et al. 2013), birth defects (Feldman et al. 2012), liver diseases (McCullough et al. 2011), and neuropsychiatric disorders (Rivas et al. 2013).

Effects of alcohol range from sedation, characterized by decreased awareness and ability to function, to addiction. The term “alcoholic” was introduced by the physician Magnus Huss in 1849 to describe alcohol addiction and can be defined as persistence of excessive drinking over a long period of time despite adverse health effects and disruption of social relations. Not all individuals who consume alcohol become alcoholics. Vulnerability to develop addiction depends on genetic, physiological and environmental conditions. Sustained alcohol intake can lead to functional alcohol tolerance, which enables increased alcohol consumption with fewer signs of intoxication. Alcohol tolerance allows escalation of drinking and eventually development of addiction. Alcohol “dependence” generally refers to physiological addiction, when cessation of alcohol intake precipitates withdrawal reactions, which range from anxiety and shakiness to severe complications, such as seizures and delirium tremens. The term “alcohol preference” refers to selectively bred strains of laboratory rats and mice that either prefer or avoid alcohol consumption. Alcohol preferring rodents voluntarily consume greater amounts of alcohol than non-preferring animals and have been studied extensively as models for alcohol addiction in humans.

Sensitivity to alcohol exposure varies among individuals within and across populations. From a genetics perspective, susceptibility to the inebriating effects of alcohol and alcohol addiction can be viewed as quantitative traits that result from the cumulative effects of multiple segregating genes and their interactions with the environment. Thus, there is no single locus that predisposes to alcohol abuse and dependence disorders, but rather many variants and their interactions with each other and the environment underlie alcohol-related phenotypes (Ducci and Goldman 2008; Morozova et al. 2012; Edenberg 2013). Disentangling the genetic and environmental contributions that shape alcohol-related phenotypes is complicated, because in human populations neither the genetic background nor the environment can be controlled precisely, results of excessive alcohol consumption are diverse, ranging from sedation to addiction and often confounded by neuropsychiatric conditions, and different studies have utilized different measurements to document alcohol-related phenotypes, such as frequency of drinks, total amount of alcohol consumed per drinking session, age at first drink, and withdrawal symptoms. This review integrates insights obtained from different model systems as well as human population studies to provide a comprehensive overview of the genetic factors that mediate sensitivity to alcohol.

## Alcohol metabolism

**Fig. 1:**
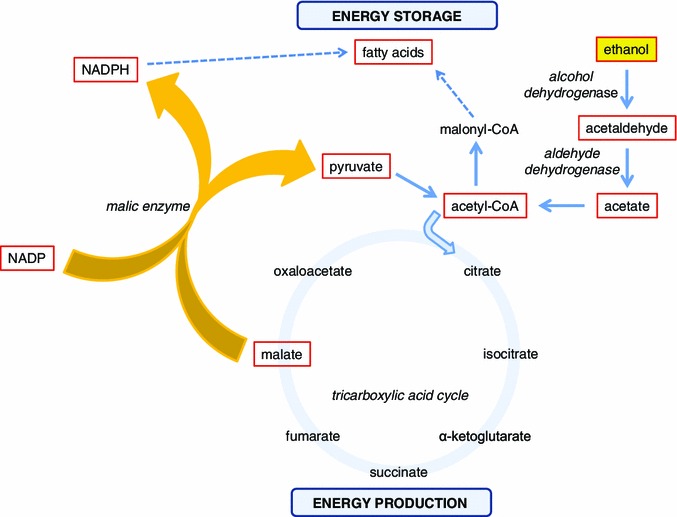
Schematic representation of the effects of ethanol on intermediary metabolism. The malic enzyme reaction is highlighted as a central mechanism that, in the presence of high ethanol concentrations, switches the flow of intermediary metabolism from energy production to energy storage by generating precursors for fatty acid biosynthesis. Central components in this pathway are illustrated in *red bordered boxes*

The rate at which alcohol is metabolized and the nature and fate of its degradation products are important factors that determine its physiological effects. The primary pathway of ethanol metabolism in liver, and to a lesser extent in the stomach and intestinal tract, involves conversion of ethanol to acetaldehyde by alcohol dehydrogenase (ADH; Fig. 1), with acetaldehyde playing a major role in mediating aversive and rewarding effects of ethanol. Acetaldehyde is oxidized further to acetate by aldehyde dehydrogenase (ALDH; Fig. 1). The human genome contains five *ADH* classes with a total of seven closely related genes located on chromosome 4q. Candidate gene approaches, as well as family-based linkage studies together with genome-wide association studies (GWAS), have implicated many variants in and around the seven *ADH* genes that contribute to alcohol dependence or alcohol-related traits, including alleles of *ADH1A*, *ADH1B*, *ADH1C*, *ADH2* and *ADH4*. While there are 18 genes encoding members of the ALDH enzyme family, only ALDH2 plays a major role in oxidizing acetaldehyde in the liver (Edenberg 2013).

Alcoholism is less common in East Asian and Polynesian populations than in European populations, due to protective *ADH* and *ALDH* alleles. A variant of *ADH1B* (ADH1B*2; Arg47His), which occurs primarily in Asian and Polynesian populations, shows 100-fold higher enzymatic activity (Thomasson et al. 1995; Mulligan et al. 2003; Birley et al. 2009; Bierut et al. 2012; Hurley and Edenberg 2012), thereby attenuating the development of tolerance. Furthermore, the ALDH2*2 allele (Glu504Lys), which is also common in East Asia, is associated with aversive reactions to alcohol consumption, such as facial flushing, hypotension, headaches and nausea (Edenberg 2007). Polymorphisms in two other enzymes, ALDH1A1 (Lind et al. 2008) and ALDH1B1 (Linneberg et al. 2010), have also been associated with alcohol consumption in Finnish and Danish populations, respectively.

Studies on the Wistar-derived UChB line of rats, which were bred for high ethanol intake, replicated the protective ALDH2 phenotype observed in Asian populations following intravenous injection of an adenoviral vector with an *Aldh2* antisense gene. This led to an 85 % decrease in ALDH2 activity in the liver and inhibited voluntary ethanol consumption up to 50 % (Ocaranza et al. 2008). Furthermore, studies with an adenoviral vector containing a multiple expression cassette showed that simultaneous increase of ADH and decrease of ALDH2 activities in the liver dramatically reduced voluntarily ethanol consumption of alcohol-dependent animals (Rivera-Meza et al. 2012).

Additional insights in the metabolism of alcohol come from studies on the fruit fly *Drosophila melanogaster*. Fruit flies encounter ethanol in their natural habitat, since larvae feed on fermented food sources, which provide substrates for lipid synthesis (Geer et al. 1985). They show preference for ethanol containing food over non ethanol containing food in laboratory experiments because of the caloric value of ethanol (Pohl et al. 2012). Both *Adh* and *Aldh* contribute to ethanol resistance in Drosophila (Fry and Saweikis 2006). There are two *Adh* alleles, designated Slow (*AdhS*) and Fast (*AdhF*) based on their electrophoretic mobility, that differ by a single amino acid (McDonald et al. 1980). Fast homozygotes have a higher level of enzymatic activity than Slow homozygotes and higher tolerance to alcohol in laboratory toxicity tests (McKenzie and McKechnie 1978). The physiological and behavioral aspects of ethanol in *D. melanogaster* have been reviewed (Kaun et al. 2012; Devineni and Heberlein 2013).

In addition to ADH, mutational analysis, artificial selection experiments, and analysis of transcript profiles following exposure to ethanol identified malic enzyme as a critical metabolic switch in response to alcohol exposure (Morozova et al. 2009). Malic enzyme converts malate, a Krebs cycle intermediate, to pyruvate, while generating NADPH. This provides the basic building blocks for the biosynthesis of fatty acids, thus switching the metabolism of energy-rich ethanol from combustion to lipid biosynthesis (Fig. 1). A similar pathway operates in people, where heavy drinking results in fatty liver disease, which can lead to inflammation and progress towards cirrhosis. Indeed an association study on the Framingham Offspring cohort revealed seven intronic SNPs in the cytoplasmic *malic enzyme 1* gene that were associated with variation in cocktail drinking (Morozova et al. 2009).

In contrast to the liver, pathways for ethanol metabolism in the brain have been difficult to elucidate (Tabakoff and Hoffman 2013). Studies on rat brain homogenates suggest that ethanol metabolism proceeds here via catalase and cytochrome P450 (CYP2E1), which inactivate about 60–70 % and about 20 % of ethanol, respectively, via oxidation (Zimatkin et al. 2006). Studies on the UChB rats in which an antisense construct against catalase was delivered via a lentiviral vector in the ventral tegmental area (VTA) led to decreased levels of ALDH activity and abolished voluntary ethanol consumption (Karahanian et al. 2011; Tampier et al. 2013). When ADH was delivered into the VTA via a lentiviral vector alcohol intake increased significantly (Karahanian et al. 2011). Insights derived from these findings may contribute to the development of new strategies for the treatment of alcohol dependence in people.

## Physiological effects of alcohol

Ethanol readily crosses the blood brain barrier and excessive alcohol consumption exerts both short-term and long-term effects on the nervous system. Acute effects are characterized by agitation and sedation, whereas long-term effects result in induction of tolerance and addiction. Different animal models can be utilized to optimally study these two aspects of alcohol consumption. Whereas alcohol-induced agitation and sedative effects of alcohol contribute in a major way to the socioeconomic cost of alcohol abuse (e.g., violence and drunk driving incidents, respectively), a vast proportion of human population studies has focused on genetic susceptibility to addiction. We will first demonstrate how *D. melanogaster* can serve as a powerful model to investigate the genetic basis of alcohol-induced sedation and how rodent models can be used to study alcohol addiction. We will then integrate studies from these model organisms with results from genetic studies on human populations.

### A Drosophila model for alcohol sensitivity

Drosophila provides a powerful genetic model for studies on alcohol sensitivity, because large numbers of genetically identical individuals can be grown rapidly under controlled environmental conditions, and a wealth of community resources for genetic studies is available. Furthermore, flies exposed to ethanol undergo physiological and behavioral changes that resemble human alcohol intoxication. Assays have been developed to precisely quantify sensitivity to alcohol by measuring alcohol-induced knock-down time (Weber 1988). Low concentrations of ethanol stimulate locomotor activity, whereas high concentrations induce an intoxicated phenotype that shows marked similarities to human alcohol intoxication, characterized by locomotor impairments, loss of postural control, sedation and immobility (Singh and Heberlein 2000; Wolf et al. 2002). Ethanol provides an ecologically relevant chemical signal for flies to locate food and oviposition sites. Flies are attracted to low concentrations of ethanol via the olfactory system, but are repelled by high concentrations and this avoidance is mediated via gustatory perception (Devineni and Heberlein 2009). Conditions under which flies show preferential intake of ethanol have been reported and it has been proposed that such conditions could mimic aspects of addiction (Devineni and Heberlein 2009; Kaun et al. 2012; Peru y Colón de Portugal et al. 2013). Repeated exposure to ethanol induces tolerance in flies, similar to humans (Scholz et al. 2000).

**Fig. 2:**
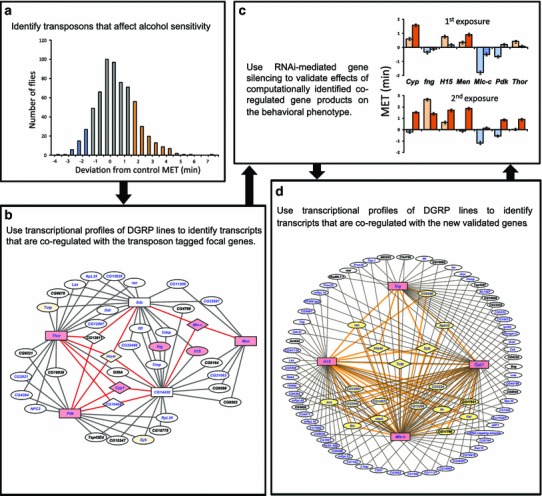
A strategy for the construction of genetic networks that underlie alcohol sensitivity in *Drosophila melanogaster*. The diagrams illustrate an iterative approach that combines *P*-element insertional mutagenesis (**a**), computational analysis of transcriptional profiles of DGRP lines (**b**, **d**) and RNAi-mediated target gene silencing (**c**) to generate a comprehensive network for alcohol sensitivity. **a** *P*-element insertional mutagenesis screen for alcohol sensitivity. *Blue and orange colored bars* designate lines with higher and lower alcohol sensitivity than control. **b** A network of co-regulated candidate genes associated with alcohol sensitivity. *Rectangles* indicate the most highly connected focal genes; genes targeted for validation studies are shown on *pink and yellow backgrounds*, and genes with annotated human orthologs are indicated in *blue font*. **c** Differences in mean elution time (MET) among RNAi lines after a first and a second exposure to ethanol. *Blue bars* indicate sensitive lines with MET lower than the control (*P* < 0.05); *orange bars* indicate resistant lines with MET higher than the control (*P* < 0.05); *gray bars* indicate no significant difference in MET. **d** An expanded network for alcohol sensitivity derived from highly interconnected genes from *panel* **b** is shown in *pink boxes*. Genes connected to two focal genes are shown at the periphery of the circle in *white ovals* and connected by *gray lines*. Genes interconnected by three and more networks are indicated on a *yellow background* and connected by *orange lines*. *Diamond shapes* indicate genes connected to all four focal genes. Genes with annotated human orthologs are indicated in *blue*. Modified from Morozova et al. (2011)

Studies on flies have utilized two complementary strategies: single gene approaches aimed at the characterization of individual candidate genes identified through mutagenesis screens and systems genetics approaches to identify genetic networks associated with alcohol sensitivity. A *P*-element insertional mutagenesis screen for alcohol sensitivity revealed that almost 30 % of the *P*-element insertions tested affected the trait, indicating that a large fraction of the genome contributes to alcohol sensitivity and predicting extensive pleiotropy (Morozova et al. 2011; Fig. 2). Indeed, most of the mutations that affect alcohol sensitivity in Drosophila have pleiotropic effects on other complex traits (Harbison and Sehgal 2008; Edwards et al. 2009; Magwire et al. 2010).

The effects of several mutants associated with alcohol sensitivity have been characterized in detail. From these studies cyclic AMP, PI3K/Akt and epidermal growth factor signaling pathways emerge as evolutionarily conserved signaling pathways that affect ethanol sensitivity in both flies and mammals (Eddison et al. 2011; Park et al. 2000; Neasta et al. 2011). Mutants associated with cyclic AMP signaling and alcohol sensitivity include *cheapdate*, an allele of *amnesiac* (Feany and Quinn 1995; Moore et al. 1998), the calcium/calmodulin-dependent adenylate cyclase *rutabaga* (Moore et al. 1998) and cyclic AMP-dependent protein kinase (Park et al. 2000). *Arouser* encodes a predicted adaptor protein homologous to the mammalian Epidermal Growth Factor Receptor Substrate 8 (Eps8) family (Eddison et al. 2011) and *happy hour* encodes a member of the Ste20 family of kinases that negatively regulate epidermal growth factor/extracellular signaling-related kinases (Corl et al. 2009).

Several synaptic neurotransmitter pathways have also been implicated in alcohol sensitivity, including signaling through dopamine (Bainton et al. 2000; Kong et al. 2010), the GABA_B_R1 receptor (Dzitoyeva et al. 2003), neuropeptide F (Wen et al. 2005) and octopamine (Scholz et al. 2000; Rothenfluh and Heberlein 2002; Guarnieri and Heberlein 2003). In addition, the postsynaptic density protein Homer plays a role in regulation of synaptic plasticity and neuronal development (Foa and Gasperini 2009) and alcohol sensitivity, in both flies and mammals (Urizar et al. 2007; Cozzoli et al. 2009). Other mutants include *slowpoke*, which encodes a large-conductance calcium-activated potassium channel (Cowmeadow et al. 2005, 2006), and the transcription factors *hangover* (Scholz et al. 2005) and *dLmo/Beadex*. The mouse ortholog of *dLmo/Beadex*, *Lmo3*, also affects alcohol sensitivity (Lasek et al. 2011).

While “one-gene-at-a-time” studies can provide important information on the contributions of individual genes and evolutionarily conserved pathways to alcohol sensitivity, it is becoming increasingly clear that complex phenotypes are emergent features of dynamic networks of interacting genes (Ayroles et al. 2009; Morozova et al. 2012). Systems genetics approaches can generate insights in the relationship between DNA sequence variants, variation in transcript abundance and variation in organismal phenotype, gene-gene interactions and the effects of genetic or environmental perturbations on the composition of genetic networks (Mackay 2014).

Combining expression microarray analysis with artificial selection from a diverse base population identified candidate genes associated with alcohol sensitivity (Morozova et al. 2007). In addition, transcript profiles of isogenic Canton-S flies also identified differentially expressed genes following repeated alcohol exposures (Morozova et al. 2006). Acute exposure to concentrated ethanol vapor resulted in rapid down-regulation of genes affecting olfaction and up-regulation of genes encoding biotransformation enzymes. Subsequent development of tolerance was accompanied by changes in expression of transcriptional regulators, proteases and metabolic enzymes, including enzymes associated with fatty acid biosynthesis (Morozova et al. 2006).

Further insights in the complex underlying genetic architecture of alcohol sensitivity were obtained by capitalizing on whole genome transcriptional profiles of a subset of 40 lines from the *D. melanogaster* Genetic Reference Panel (Ayroles et al. 2009), a wild-derived population of inbred lines with fully sequenced genomes (Mackay et al. 2012). Variation in alcohol sensitivity and induction of tolerance was associated with variation in transcript abundance levels and significantly associated transcripts could be grouped in modules of genetically co-regulated transcripts (Morozova et al. 2009). Modules associated with acute alcohol sensitivity were distinct from those associated with induction of tolerance, indicating that the genetic architectures that underlie the immediate response to ethanol exposure and induction of tolerance are distinct. Notably, genes implicated in nervous system function were associated with natural variation in tolerance development but not with acute ethanol exposure. Connectivity of focal genes within these modules could be validated through *P*-element mediated mutagenesis, followed by measurements of transcript abundances of connected genes within the module, illustrating the power of complementary single gene and systems approaches (Morozova et al. 2009).

A reverse approach is to identify *P*-element mutations that affect sensitivity or resistance to alcohol exposure as focal genes and derive computational networks of covariant transcripts centered on these focal genes. The contributions of computationally recruited genes to alcohol sensitivity can then be confirmed by RNAi-mediated inhibition of their expression. Furthermore, these genes can then, in turn, serve as focal genes to expand the computationally derived networks by iteration, allowing a gradual buildup of the network with simultaneous functional validation (Morozova et al. 2011; Fig. 2). At least 60 % of Drosophila genes have human orthologs and these orthologs can be superimposed on the genetic networks from Drosophila. This can provide a stepping stone for the identification of candidate genes associated with alcohol-related phenotypes in human populations (Morozova et al. 2009).

### Rodent models for alcohol dependence

Several animal systems have been used to model different aspects of human alcohol-related phenotypes, including preference for alcohol consumption and withdrawal (Bennett et al. 2006). Rodents are suitable models for studies on genetic susceptibility for alcohol dependence, since the organization of regions of the nervous system that mediate addiction is conserved among mice, rats and humans, including the projection from the VTA to the nucleus accumbens and the mesolimbic dopaminergic projection to the forebrain. Furthermore, neurotransmitter systems that regulate this reward pathway are similar. Studies on rodents show that different genes are associated with alcohol consumption and withdrawal effects, similar to the different genetic architectures for inebriation and induction of tolerance seen in the Drosophila model.

Single gene studies in mice have implicated more than 70 candidate genes in alcohol-related phenotypes (Crabbe et al. 2006). These include genes encoding calcium-stimulated adenylate cyclase and protein kinase A (Maas et al. 2005), calcium- and voltage-gated potassium channels (Blednov et al. 2003a; Martin et al. 2008), protein kinase C (Wand et al. 2001; Bowers et al. 2006), neuropeptide Y (Thiele et al. 1998; Thorsell 2007), and proteins involved in GABA neurotransmission (Blednov et al. 2003b; Boehm et al. 2004), dopamine and serotonin signaling (Fadda et al. 1991; Hall et al. 2003; Kelai et al. 2003; Crabbe et al. 2006; Bilbao 2013).

A large number of studies aimed at identifying genes that contribute to variation in alcohol-related phenotypes have relied on gene mapping strategies. At least 24 quantitative trait loci (QTL) have been identified in the mouse genome (Crabbe et al. 1999) and four genomic regions were found in rat (Saba et al. 2011). Meta-analysis of QTL mapping across eight different studies on murine alcohol consumption provided strong support for four QTL regions located on mouse chromosomes 2, 3, 4 and 9 (Belknap and Atkins 2001). Candidate genes have been identified within these QTL regions, including genes encoding a multiple PDZ domain protein (Mpdz) on mouse chromosome 4 (Fehr et al. 2002) and *syntaxin binding protein 1* on mouse chromosome 2 (*Stxbp1*; Fehr et al. 2005) and genes for neuropeptide Y, α-synuclein and corticotrophin-releasing factor receptor 2 in rats (Spence et al. 2009). However, evidence that links candidate genes within QTL regions causally to the phenotype remains difficult to obtain.

Studies that integrate QTL mapping and gene expression analyses have been used both in mice and rats to facilitate the identification of candidate genes that contribute to linkage signals. Previously, two independent studies identified alcohol dependence QTLs on human chromosome 1q (Dick et al. 2002; Hill et al. 2004), and additional studies also provided support for association of markers on chromosome 1q with alcoholism (Aragaki et al. 1999; Turecki et al. 1999; Guerrini et al. 2005). QTL mapping studies in mice revealed 15 differentially expressed genes associated with alcohol withdrawal on chromosome 1, syntenic with human chromosome 1q. Among these genes *Kcnj9* appeared the most promising candidate gene. *Kcnj9* encodes GIRK3, which is a subunit of inward rectifying K^+^ channels that mediate inhibitory effects of G_i/o_ coupled receptors (Kozell et al. 2009). In addition, two independent gene profiling experiments identified candidate genes for alcohol consumption QTL within the rat chromosome 4 region (Carr et al. 2007; Liang et al. 2010), including *Akr1b1*, *Copg2*, *Dgki*, *Grid2*, *Npy*, *Plxna4*, *Ppm1k*, *Qdpr*, *Scap2*, *Snca*, *Snx10* and *Spr,* which are involved in dopamine and serotonin signaling, protein trafficking and signal transduction. Furthermore, a transcriptome meta-analysis identified 20 *cis*-regulated candidates for alcohol preference QTL on mouse chromosome 9, including *Carm1*, *Cryab*, *Pknox2* and *Scn4b* (Mulligan et al. 2006).

A combination of QTL mapping and microarray analyses in rats identified candidate genes associated with alcohol consumption and among them genes that were differentially expressed in brains as a result of alcohol intake. These genes implicated pathways associated with GABA release, activation of dopamine neurons and postsynaptic GABA receptor trafficking (Tabakoff et al. 2009). A similar strategy applied to high and low alcohol-consuming mice identified *Gnb1* localized within a QTL region on chromosome 4 as a candidate gene, since it was also differentially expressed in brains (Saba et al. 2011). *Gnb1* codes for the β1 subunit of guanine nucleotide binding proteins. A subsequent comparative genomics approach to study relationships among differentially expressed genes that contribute to alcohol drinking in rats, mice and humans revealed a neural signaling pathway that encompasses both presynaptic and postsynaptic elements of GABA signaling and includes Gβ1. These findings highlight cross-species similarities in genes and pathways that underlie alcohol consumption in animal models and humans and show that neural signaling pathways feature prominently in determining susceptibility to alcohol intake.

Mulligan et al. (2011) used a systems network approach to identify in different brain regions modules of co-regulated genes that showed changes in expression associated with acute drinking. Gene ontology enrichment analyses showed that the modules represented different physiological processes, functional groups, and cell types. Most genes with altered expression were specific for a brain region, suggesting that the response to acute alcohol exposure is cell type specific. Nevertheless, meta-analysis across all brain regions identified a subset of 42 alcohol responsive genes that were shared across multiple brain regions. Several of these genes, including *Hba*-*a1*, *Hbb*-*b1*, *Fth1*, *Gstm1*, *Mt2*, *Nfkbia*, *Park7*, *Pltp*, *Prkcz*, *Qdpr* and *Scn4b*, were associated with responses to ethanol in different species or were identified in multiple independent studies.

Combining transcript analyses with network and pathway analyses can provide functional context for candidate genes and help to prioritize them for functional validation. This strategy was successfully implemented by Blednov et al. (2012), who selected null mutant mice for six previously identified candidate genes related to peripheral immune and inflammatory signaling, encoding beta-2-microglobulin (*B2m*), cathepsin S (*Ctss*), cathepsin F (*Ctsf*), interleukin 1 receptor antagonist (*Il6*), CD14 molecule (*Cd14*) and interleukin 6 (*Il6*), and showed that all six mutants displayed changes in alcohol consumption. In contrast to previous studies that identified genes involved in neural signaling, none of these genes had previously been associated with alcohol-related phenotypes.

A transcriptional profiling study, which examined changes in transcript abundances in liver and prefrontal cortex in mice that voluntarily consume alcohol, identified a network of genes in prefrontal cortex associated with dopaminergic signaling as well as immune function. Orthologs of these genes include *Drd1*, *Drd2*, *Fos*, *Fosb* and *Ppp1r1b*, implicated previously in ethanol and drug addiction (Osterndorff-Kahanek et al. 2013). In liver, transcriptional changes were more numerous and comprised a genetic network associated with drug metabolism and glutathione depletion (Osterndorff-Kahanek et al. 2013). These studies indicate a link among ethanol intake, dopamine signaling and immune responses. The connection with the immune system is of interest, since bacterial lipopolysaccharide can leak from the gut as a result of chronic alcohol abuse and activate the immune system (Mandrekar and Szabo 2009). This bacterial endotoxin also increases alcohol consumption in mice (Blednov et al. 2011).

Following chronic alcohol exposure, removal of alcohol produces a range of withdrawal symptoms, which increase motivation to seek and consume alcohol. Withdrawal symptoms are a hallmark of physiological dependence and include convulsions, motor abnormalities, anxiety and irritability. Symptoms are qualitatively similar across species, but range in severity across individuals and last up to 48 h. Severity of alcohol withdrawal and alcohol preference drinking are genetically negatively correlated; genotypes that drink a lot of ethanol are genetically predisposed to have low withdrawal severity, and vice versa (Metten et al. 1998). Seminal studies by Buck and colleagues (Fehr et al. 2002; Shirley et al. 2004) identified the *Mpdz* gene within a QTL region on mouse chromosome 4 as a causal gene with large effects on alcohol and barbiturate withdrawal. MPDZ interacts physically with GABA_B_ receptors (Balasubramanian et al. 2007), 5-HT_2C_ receptors (Becamel et al. 2001) and postsynaptic density-associated GTPase-activating protein (SynGAP), which binds to the NMDA receptor 2B subunit/Ca^2+^-calmodulin kinase (MPDZ/NR2B/CaMKII) complex (Krapivinsky et al. 2004; Kim et al. 2005). Thus, MPDZ could modulate ethanol withdrawal symptoms by regulating GABA_B_ and/or 5-HT_2C_ receptor-mediated neurotransmission (Chen et al. 2011a). Using expression of the immediate early gene *c-Fos* as a marker for neuronal activation, Chen et al. (2008) found that animals congenic for the chromosome 4 QTL containing the *Mpdz* gene exhibited significantly less ethanol withdrawal-associated neuronal activation within the basal ganglia than background controls. This effect was particularly evident in the caudolateral region of the substantia nigra pars reticulata, where bilateral electrolytic lesions resulted in attenuation of the severity of ethanol withdrawal symptoms (Chen et al. 2011b). The mouse chromosome 4 QTL is syntenic with a region on human chromosome 9p. Indeed, human MPDZ has also been implicated in alcohol dependence (Karpyak et al. 2009, 2012; Buck et al. 2012).

### Human genetic studies of alcohol-related phenotypes

Human genetic studies have identified polymorphisms associated with alcohol dependence in genes that comprise various neurotransmitter signaling pathways, including dopaminergic (e.g., *MAOA*, *COMT*, and *DRD2*, *ANKK1*, *TTC12* and *NCAM1*; Kohnke et al. 2005; Yang et al. 2008; Tikkanen et al. 2009; Hendershot et al. 2011); serotonergic (e.g., *5*-*HTT*, *SLC6A4* and *HTR3A*, *HTR3B*, *HTR1B*; van der Zwaluw et al. 2010; Cao et al. 2013; Seneviratne et al. 2013); GABAergic (e.g., *GABRA1*, *GABRA2* and *GABRG1*; Agrawal et al. 2006; Dick and Bierut 2006; Enoch 2008), glutamatergic (*GRM8*; Chen et al. 2009), and cholinergic systems (e.g., *CHRM2* and *CHRNA5*, *CHRNB2*; Luo et al. 2005; Ehringer et al. 2007; Wang et al. 2009); opioid receptors (e.g., prodynorphin; *PDYN*; Flory et al. 2011, *OPRM1*, *OPRD1* and *OPRK1*; Ray and Hutchison 2004; Zhang et al. 2008; Ashenhurst et al. 2012), and tachykinin receptor 3 (*TACR3*; Foroud et al. 2008).

In recent years, transcriptional profiling and GWAS also reported candidate genes associated with risk for alcohol dependence (for recent reviews, see Spanagel et al. 2010; Morozova et al. 2012; Rietschel and Treutlein 2013). However, with the exception of the large effects contributed by variation at *ADH1B* and *ALDH2* in Asian populations (Edenberg 2007; Hurley and Edenberg 2012), there is little consistency across studies. Nevertheless, one gene encoding cadherin 13 (*CDH13*) was replicated in four independent studies among all SNPs that were significant at a nominal *P* value (Johnson et al. 2006; Liu et al. 2006b; Treutlein et al. 2009; Lind et al. 2010). In addition, several risk loci for alcohol dependence and consumption have been detected with samples from large datasets, such as the Collaborative Studies on the Genetics of Alcoholism (COGA), the Study of Addiction: Genetics and Environment (SAGE) and the Australian Twin-family study of alcohol use disorder (OZ-ALC). These include Rho GTPase-activating protein 28 (*ARHGAP28*), CUB and Suchi multiple domain 1 and 2 (*CSMD1* and *CSMD2*), Catenin delta 2 (*CTNND2*), Kv channel interacting protein 1 (*KCNIP1*), Neuronal PAS domain protein 3 (*NPAS3*), Protein tyrosine phosphatase, receptor type D (*PTPRD*) and Usher syndrome 2A (*USH2A*; reviewed by Morozova et al. 2012). The lack of replication across GWAS could be explained by heterogeneity of study populations with different allele frequencies and epistatic interactions as well as different measurements of alcohol consumption. For example, in a GWAS study on individuals from the COGA dataset, Dick et al. (2008) found a gene associated with alcohol dependence on chromosome 7 encoding ACN9 homolog (*ACN9*), which is involved in gluconeogenesis (Dennis and McCammon 1999). However, a recent analysis of 14 genes involved in alcohol metabolism and oxidation revealed no association between polymorphisms in the *ACN9* gene when blood and breath alcohol metabolites were used as phenotypic measurements. Instead, they found significant associations with a SNP in the promoter of *CYP2E1* and SNPs in catalase (*CAT*), beta-enolase (*ENO3*) and glutamic-oxaloacetic transaminase 1 (*GOT1*; Lind et al. 2012).

To increase statistical power and ability to detect novel risk loci several groups have conducted meta-analyses using data from the available COGA, SAGE and OZ-ALC GWAS for alcohol dependence (Zuo et al. 2011, 2013a, b; Wang et al. 2011a, b). Interestingly, meta-analyses using similar datasets identified different risk alleles. One study which reanalyzed the COGA and SAGE datasets identified thrombospondin type I domain containing 7B (*THSD7B*), *KIAA0040*, nardilysin (N-arginine dibasic convertase, *NRD1*), PBX/knotted 1 homeobox 2 (*PKNOX2*), anaplastic lymphoma receptor tyrosine kinase (*ALK*), cancer susceptibility candidate 4 (*CASC4*) and semaphorin 5A (*SEMA5A*). Three of these genes, *THSD7B*, *KIAA0040* and *NRD1*, were further replicated in OZ-ALC samples (Wang et al. 2011b). *PKNOX2* and *KIAA0040* genes have been also associated with alcohol dependence in other studies (Bierut et al. 2010; Chen et al. 2011c; Zuo et al. 2012).

A family-based association analysis for alcohol dependence that utilized both COGA and the OZ-ALC samples conducted by the same group (Wang et al. 2011a) found several additional genes associated with alcohol dependence, including endothelin receptor type B (*EDNRB*), Down syndrome cell adhesion molecule like 1 (*DSCAML1*), TCDD-inducible poly(ADP-ribose) polymerase (*TIPARP*), monoamine oxidase A (*MAOA*), Na^+^/K^+^ transporting ATPase interacting 2 (*NKAIN2*) and Usher syndrome 2A (*USH2A*), among which *USH2A* and *MAOA* genes have been previously associated with alcohol dependence (Johnson et al. 2006; Tikkanen et al. 2009; Heath et al. 2011).

A different set of studies, using GWAS and eQTL analyses, also reanalyzed data from the COGA, SAGE, and the OZ-ALC GWAS for alcohol dependence and identified several replicable risk regions for alcohol dependence (Zuo et al. 2011, 2013a, b). Candidate genes within these regions included Plant HomeoDomain finger protein 3-protein tyrosine phosphatase type IVA, member 1 (*PHF3*-*PTP4A1*; Zuo et al. 2011), Na^+^/K^+^ transporting ATPase interacting 1-serine incorporator 2 (*NKAIN1*-*SERINC2*; Zuo et al. 2013a); and importin 11-5-hydroxytryptamine (serotonin) receptor 1A (*IPO11*-*HTR1A*), associated with both alcohol and nicotine codependence (Zuo et al. 2013b). In addition, expression of the *PHF3*, *PTP4A1*, *NKAIN1*, and *SERINC2* transcripts was significantly correlated with expression of numerous genes implicated in addiction, including those in dopaminergic (*DRD1*, *DRD2*, *NCAM1*, *PPP1R1B*) serotonergic (*HTR1B*, *HTR2A*), cholinergic (*CHRNA3*, *CHRNB2*), GABAergic (*GABRA1*, *GABRA2*, *GABRG2*), glutamatergic (*GAD1*, *GRIK3*, *GRIN2C*), neuropeptide Y (*NPY1R*, *NPY5R*) and opioid systems (*OPRD1*, *OPRM1*, *POMC*) (Zuo et al. 2011, 2013a) as well as genes associated with alcohol metabolism (*ADH4*, *ADH5*, *ADH6*). Moreover, injection of ethanol into the brains of mice resulted in altered expression of *Ipo11* and *Ptp4a1* (Kerns et al. 2005) and *Nkain1*, *Nrd1* and *Phf3* were differentially expressed in brains of alcohol-drinking mice (Mulligan et al. 2006).

Two recent studies on the same large datasets used the maximum number of alcoholic drinks consumed in a 24-h period (MaxDrinks) as phenotypic measurement (Kapoor et al. 2013; Pan et al. 2013). Both studies found different genes from those identified by previous meta-analyses and the results from these two studies showed little, if any, overlap.

One study which used the MaxDrinks criterion based on samples from the COGA and SAGE datasets found only two associated genes with genome-wide significance, LIM domain only 1 (*LMO1*) and phospholipase C-like 1 (*PLCL1*) (Kapoor et al. 2013). As mentioned earlier, orthologs of the LMO gene family regulate behavioral responses to ethanol in *D. melanogaster* and mice (Lasek et al. 2011). In addition, nominally significant SNPs were identified in autism susceptibility candidate 2 (*AUTS2*), inaD-like (*INADL*), chromosome 15 open reading frame 32 (*C15orf32*), and huntingtin interacting protein (*HIP1*) genes. These genes were also implicated in alcohol consumption previously (Heath et al. 2011). The Drosophila *AUTS2* ortholog also contributes to alcohol sensitivity (Schumann et al. 2011).

A separate study on the COGA, SAGE and OZ-ALC datasets which also used MaxDrinks as the phenotype identified different candidate genes, including shugoshin-like 1 (*SGOL1*), nuclear receptor subfamily 4, group A, member 2 (*NR4A2*) gene and DTW domain containing 2 (*DTWD2*), *N*-deacetylase/*N*-sulfotransferase (heparan glucosaminyl) 4 (*NDST4*), potassium voltage-gated channel, Shab-related subfamily, member 2 (*KCNB2*) and DOPA decarboxylase (*DDC*; Pan et al. 2013).The *NR4A2* gene has been previously implicated in alcohol dependence in Mexican Americans (Wei et al. 2012), *DDC* has been associated with alcohol consumption in women (Agrawal et al. 2011) and *SGOL1* has been implicated in alcohol dependence (Edenberg et al. 2010).

Several of the candidate risk genes for alcohol dependence identified in these studies contribute to alcohol-related behaviors in animal models. Orthologs of *PKNOX2*, *SEMA5A,* *USH2A*, *DSCAML1, NR4A2, DDC, SGOL1, DTWD2* and *NDST4* show differential expression in brains from alcohol-drinking mice compared to controls; (Mulligan et al. 2006, 2011; Wolstenholme et al. 2011). *MAOA* and *DSCAML1* orthologs were also differentially expressed in alcohol preferring rats (Rimondini et al. 2002; Rodd et al. 2008) and a *DSCAML1* ortholog was also found in flies selected for alcohol sensitivity (Morozova et al. 2007).

Zhao et al. (2012) performed a cross-species meta-analysis by ranking genes differentially expressed in mouse brains in response to ethanol (Kerns et al. 2005; Mulligan et al. 2006) based on orthologs implicated in alcohol-related phenotypes across multiple species, including humans (Reich et al. 1998; Mayfield et al. 2002; Kuo et al. 2006; Prescott et al. 2006; Hodgkinson et al. 2008); *C. elegans* (Kwon et al. 2004) and *D. melanogaster* (Morozova et al. 2006, 2007) and identified *BDNF*, *GABRA2*, *GABRB1*, *MPDZ*, *NPY* and *NPY2R* among the top ranked genes.

**Fig. 3:**
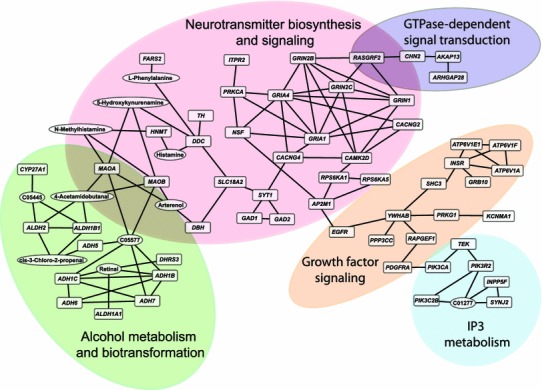
A comprehensive genetic network for alcohol-related phenotypes. The network is derived from a meta-analysis that incorporates candidate genes from GWAS and differentially expressed genes from transcriptional profiling studies on humans and model organisms (supplementary Table 1). *Ovals* indicate metabolites that interconnect gene products. The network was constructed using the R-Spider algorithm in the bioprofiling.de web portal (Antonov 2011). This analysis tool incorporates data for ∼4,000 human genes and combines signaling and metabolic pathways from Reactome and KEGG databases to determine whether interactions between the input genes are greater than expected by chance. The network is built by connecting genes with known interactions in the two databases. The significance of the network is tested by Monte Carlo simulations, in which the same number of randomly selected genes is used to form the null distribution of the size of the network. The network presented here consists of 58 interconnected candidate genes (*P* < 0.005). When one missing gene is allowed (i.e., a gene that does not contain polymorphisms associated with phenotypic variation), the network can be expanded to encompass 258 candidate genes (*P* < 0.03)

Using a similar approach we compiled findings from several transcriptional profiling studies that have identified differentially expressed genes from alcohol-related studies on *D. melanogaster* (Morozova et al. 2006, 2007, 2009, 2011; Urizar et al. 2007; Awofala 2010; Kong et al. 2010); from alcohol-related expression studies done on mice (Xu et al. 2001; Daniels and Buck 2002; Tabakoff et al. 2003; Hitzemann et al. 2004; Saito et al. 2004; Treadwell and Singh 2004; Kerns et al. 2005; MacLaren et al. 2006; Mulligan et al. 2006, 2011; Saba et al. 2006; Wang et al. 2007; Denmark and Buck 2008; Wolstenholme et al. 2011), and transcriptional profiling data on rats (Rimondini et al. 2002; Edenberg et al. 2005; Worst et al. 2005; Carr et al. 2007; Kimpel et al. 2007; Rodd et al. 2008), and identified human orthologs. In addition, we analyzed six published transcriptional profiling data sets performed on different areas of postmortem human brains and also included candidate genes for alcohol-related phenotypes from the HuGE Navigator database (Lewohl et al. 2000; Mayfield et al. 2002; Sokolov et al. 2003; Iwamoto et al. 2004; Flatscher-Bader et al. 2005; Liu et al. 2006a; Guo et al. 2009). Furthermore, we integrated information from GWAS, considering all candidate genes that were nominally significant (Johnson et al. 2006; Liu et al. 2006b; Dick et al. 2008; Treutlein et al. 2009; Bierut et al. 2010; Edenberg et al. 2010; Lind et al. 2010; Baik et al. 2011; Heath et al. 2011; Schumann et al. 2011; Wang et al. 2011a, b; Zuo et al. 2011, 2012, 2013a, b; Kapoor et al. 2013; Pan et al. 2013) and ranked these genes based on how many times within and between species they were replicated (supplementary Table 1). We found only seven genes that were replicated across all four species, *ARIH1*, *COPB2*, *DLG2*, *IGF2R*, *IMPA2*, *MAX* and *SHC3*, and 139 genes were replicated among any two model organisms and humans, including *ALDH1A1*, *ADD1*, *APOD*, *AUTS2*, *CAT*, *CAST*, *CRYAB*, *GABBR1*, *NFKB1*, *NRD1*, *PDIA3*, *PRKCA* and *TACR3* (supplementary Table 1). We constructed interacting ensembles between candidate genes (http://www.BioProfiling.de; Antonov 2011) and could cluster 58 genes in networks that were significantly enriched (*P* < 0.005) for alcohol metabolism and biotransformation, inositol triphosphate metabolism, neurotransmitter biosynthesis and signaling, growth factor signaling and GTPase-dependent signal transduction (Fig. 3). Among the most highly interconnected genes in this integrated network are *GRIA1* and *GRIA4*, which encode ionotropic AMPA 1 and 4 glutamate receptors, respectively, and *GRIN1*, *GRIN2B* and *GRIN2C,* which encode NMDA ionotropic glutamate receptor subunits 1, 2B and 2C, pointing at a central role for glutamatergic neurotransmission. Thus, by integrating data on alcohol-related phenotypes from GWAS and transcriptional profiling studies on both humans and model organisms it is possible to construct biologically meaningful networks of genes that contribute to alcohol dependence and identify “hub” genes as potential candidate genes for future follow-up studies.

## Alcohol-induced epigenetic gene regulation: the next frontier

Epigenetic modifications are becoming increasingly appreciated as important contributors to the effects of alcohol on regulation of gene expression. Epigenetic modifications have been implicated especially in studies on fetal alcohol spectrum disorders (Perkins et al. 2013; Resendiz et al. 2013). Epigenetic alterations include DNA methylation and histone modifications, both of which remodel chromatin structure and, thereby, influence gene expression. Recent studies established that alcohol consumption induces epigenetic alterations in various organs, including brain (Ponomarev et al. 2012; Ponomarev 2013), the gastrointestinal tract (Shukla and Lim 2013) and liver (Mandrekar 2011; Shukla and Lim 2013). In addition, excessive alcohol consumption leads to hypomethylation and promotes histone acetylation in humans and rodents (Lu et al. 2000; Wolstenholme et al. 2011; Ponomarev et al. 2012; Warnault et al. 2013; Zhang et al. 2013). Furthermore, reduction in DNA methylation by administration of a DNA methyltransferase inhibitor as well as inhibition of histone deacetylase leads to reduction in alcohol consumption in mice (Warnault et al. 2013). These observations may open the door to develop chromatin modifying agents to treat alcoholism more effectively than currently available drugs, such as disulfiram, naltrexone and acamprosate (for comprehensive reviews see Heilig and Egli 2006; Johnson 2008; Franck and Jayaram-Lindstrom 2013).

## Summary

Several GWAS and meta-analyses studies have reported a vast number of risk alleles for alcohol dependence with little overlap among studies. This is attributable to different phenotypic assessments, use of different species and different preparations, including different tissues or brain regions analyzed, and genome-by-environment interactions. The link between genotype and phenotype is likely also confounded by multidimensional gene-gene interactions, the magnitudes of which depend on allele frequencies (Mackay 2014). In addition, the genetic architectures that underlie different phenotypic manifestations of alcohol drinking behavior appear to be distinct. Nevertheless, different studies reveal different aspects of the genetic underpinnings of the physiological and behavioral effects of ethanol, while underscoring the underlying genomic complexity of the genotype-phenotype relationship. Combining and integrating information from experimentally tractable model systems with human genetic studies provides a powerful strategy to disentangle the genomic elements that contribute to alcohol-related phenotypes.

## Electronic supplementary material

Below is the link to the electronic supplementary material.

Supplementary material 1 (XLSX 76 kb)
